# Copycat suicidal attempt by a 7 year old boy after watching homicidal behavior in media: a case report

**DOI:** 10.1186/1757-1626-2-43

**Published:** 2009-01-12

**Authors:** Ahmad Ghanizadeh

**Affiliations:** 1 Shiraz University of Medical Sciences, Research Center for Psychiatry and Behavioral Sciences Hafez Hospital, Shiraz, Iran

## Abstract

**Introduction:**

Suicidal behavior in media may promote others towards suicide. No published study was found about suicidal attempt in children less than 10 years old after watching a homicidal behavior.

**Case presentation:**

This is a report of a 7 year old boy referred because he hanged himself after watching homicidal behavior of hanging in a fictional movie.

**Discussion:**

To the author's knowledge, there was no published report of copycat suicidal attempt in a 7 year old child after watching a homicidal behavior in media. This report warns about an imitative effect of movie watching of homicidal behavior on suicidal attempt.

## Introduction

Copycat suicide is a real fact [1]. Exposure to suicidal behavior in media increases copycat suicide [2]. Many studies focused on social learning theory as the theoretical basis of copycat suicide. Some human behaviors are learned through observation of model. Suicidal behavior in media precipitates those vulnerable persons towards suicide. More coverage of suicide story in media increases suicide [3]. Of course, the mood of watchers, e.g. depression, is an important factor. A study investigated the physiological reactions to a film on suicide by suicidal attempters, suicidal ideators, and non-suicidal patients. They reported that the effect of suicidal story is highly influenced by hosts characteristics [4]. Younger people have higher vulnerability to media influence [4].

All of the previous studies reported copycat and contagious suicide in adults or youth older than 10 years of age. To the authors' knowledge, no published study was found about the copycat suicide in children less than 10 years old after watching a homicidal behavior. Better understanding of the relationship between media reporting or media watching of homicidal behavior and subsequent suicidal behavior may help develop better effective strategies for suicide prevention.

This is a report of a 7 year old boy with attention deficit hyperactivity disorder referred because of suicidal attempt after watching of a fictional movie. His parent gave oral consent for publication of this report.

## Case presentation

The patient is 7 year old boy who was referred because he hanged himself after watching a fictional movie. His mother did not know the title of the movie. She reported that the movie showed four soldiers who were hanged. Of course, the soldiers were rescued immediately after hanging and there was a good outcome for them. As to the case of this study, his mother found her child had lied down in the room. There was a torn band around his neck. He was semiconscious. She realized that he hanged himself but the band was weak and his child fortunately was rescued spontaneously. The scar of the band was apparently visible on his neck. The child was interviewed. He was suffering from attention deficit disorder according to DSM-IV diagnostic criteria using KSADS Farsi version [5]. No other psychiatric co-morbid disorder such as depressive disorder or anxiety disorder was found. The child had no history of suicidal attempt or thought. Family and relatives history of suicide or suicidal attempt was negative. Nobody had heard suicidal thought from him. No general medical condition was found. There was a mild anemia few years ago when he had taken ferrous sulfate. His educational function was excellent and he never experienced educational impairment. No significant behavioral change was found. Clinically, his estimated intelligence was in the normal range.

## Discussion

To the authors' knowledge, there was no published report of copycat suicidal attempt in a 7 year old child. The focus has so far been on teenagers. There were no risk factors related to the audience such as anxiety and depression. However, he was a case of ADHD with impulsive behavior. There are limited studies that investigated the role of audiences' characteristics but those characteristics were limited to anxiety and depression [4]. This case reports that there are some other characteristics which are very common such as ADHD and impulsivity that should be considered in future studies. The role of ADHD and impulsivity for copycat suicidal attempt in very young children is an ignored research area.

There are some reports about the association of reporting of suicide and its effect on suicidal behavior of watchers [6,7]. The effects of different media such as print media, electronic media, news reporting, fictional stories, music, and the internet on suicide had been investigated. Suicide prevention strategies have not included watching of homicidal behavior [8]and it is a missed field. Association of watching homicidal behavior and suicide in audience should be studied in future studies.

This case report supports an imitative effect of movie watching of homicidal behavior on suicidal behavior in the very young children.

## Competing interests

The author declares that he has no competing interests.

## Authors' contributions

AG interviewed the patient and parent and wrote the manuscript.

## Consent

The parent gave informed consent for publication of this case report.
